# The distribution of circulating microRNA and their relation to coronary disease

**DOI:** 10.12688/f1000research.1-50.v1

**Published:** 2012-11-19

**Authors:** Jane E Freedman, Bahadir Ercan, Kristine M Morin, Ching-Ti Liu, Lulufer Tamer, Lokman Ayaz, Mehmet Kanadasi, Dilek Cicek, Ali Ihsan Seyhan, Rabia Eker Akilli, Celalettin Camci, Beyhan Cengiz, Serdar Oztuzcu, Kahraman Tanriverdi

**Affiliations:** 1 Department of Medicine, University of Massachusetts Medical School, Massachusetts, 01605, USA; 2 Department of Biostatistics, School of Public Health, Boston University, Boston, 02118, USA; 3 Department of Biochemistry, Mersin University, School of Medicine, Mersin, 33343, Turkey; 4 Department of Cardiology, Cukurova University, School of Medicine, Adana, 01110, Turkey; 5 Department of Cardiology, Mersin University, School of Medicine, Mersin, 33343, Turkey; 6 Department of Oncology, Gaziantep University, School of Medicine, Gazientep, 27310, Turkey; 7 Department of Physiology, Gaziantep University, School of Medicine, Gazientep, 27310, Turkey; 8 Sahinbey Hospital Central Laboratory, Gaziantep University, Gaziantep, 27310, Turkey

## Abstract

**Background:** MicroRNAs (miRNAs) are small RNAs that regulate gene expression by suppressing protein translation and may influence RNA expression. MicroRNAs are detected in extracellular locations such as plasma; however, the extent of miRNA expression in plasma its relation to cardiovascular disease is not clear and many clinical studies have utilized array-based platforms with poor reproducibility.

**Methods and Results:** Initially, to define distribution of miRNA in human blood; whole blood, platelets, mononuclear cells, plasma, and serum from 5 normal individuals were screened for 852 miRNAs using high-throughput micro-fluidic quantitative RT-PCR (qRT-PCR). In total; 609, 448, 658, 147, and 178 miRNAs were found to be expressed in moderate to high levels in whole blood, platelets, mononuclear cells, plasma, and serum, respectively, with some miRNAs uniquely expressed. To determine the cardiovascular relevance of blood miRNA expression, plasma miRNA (n=852) levels were measured in 83 patients presenting for cardiac catheterization. Eight plasma miRNAs were found to have over 2-fold increased expression in patients with significant coronary disease (≥70% stenosis) as compared to those with minimal coronary disease (less than 70% stenosis) or normal coronary arteries. Expression of miR-494, miR-490-3p, and miR-769-3p were found to have significantly different levels of expression. Using a multivariable regression model including cardiovascular risk factors and medications, hsa-miR-769-3p was found to be significantly correlated with the presence of significant coronary atherosclerosis.

**Conclusions: **This study utilized a superior high-throughput qRT-PCR based method and found that miRNAs are found to be widely expressed in human blood with differences expressed between cellular and extracellular fractions. Importantly, specific miRNAs from circulating plasma are associated with the presence of significant coronary disease.

## Introduction

MicroRNAs (miRNAs) are 20–26-nt single stranded RNAs that participatein the regulation of various biological functions in numerouseukaryotic lineages, including plants, insects, vertebrate, and mammals ^1– 3^. More than 850 human miRNAs have been cloned and bioinformatic predictions indicate that mammalian miRNAs can regulate approximately 30% of all protein-coding genes ^4– 6^. The expressionof many miRNAs is specific to a tissue or developmentalstage, and the miRNA expression pattern is altered during thedevelopment of several diseases ^7, 8^.

Studies measuring small numbers of miRNAs have shown their presence in circulating blood; specifically in platelets ^9^, plasma ^10^, and mononuclear cells ^11^. In studies examining specific miRNAs ^12, 13^, differential expression was noted both in hematopoietic cell lines ^13^ and between human and mouse cells ^12^. Interestingly, miRNAs have been detected in cell-free human plasma preparations ^14^. They have been found to be stable and protected from endogenous RNase activity ^14^. In addition, levels of a specific miRNA ( *miR-141*) can distinguish patients with prostate cancer from healthy controls ^14^.

Basic studies have shown that miRNAs regulate cardiac differentiation, angiogenesis, and myocyte growth ^15, 16^. Small clinical studies have also shown that levels of specific miRNA have been correlated with myocardial infarction in cardiac tissue from humans ^17^ and animal models ^18, 19^. A recent study examined one miRNA ( *miR-1*) from plasma and related it to acute myocardial infarction ^10^. In stable and unstable coronary artery disease patients, 157 miRNAs were measured from peripheral blood mononuclear cells and differential expression was found ^11^.

As shown by these studies, there are some publications about circulating miRNAs in cardiovascular disease ^9, 20^. In addition, the existing studies are restricted by incomplete evaluation of currently known miRNAs. Available arrays are constrained by incomplete miRNA coverage, issues in discriminating between closely related miRNAs, as well as ongoing discovery of new miRNAs (and the inherent lack of flexibility of the array platform). In addition, miRNA arrays have poor reproducibility when across clinical populations or in larger samples sizes ^21^. We have developed a combined method of miScript miRNA assays (Qiagen, Germantown, MD) and Dynamic Arrays (Fluidigm, South San Francisco, CA) that employs quantitative RT-PCR (qRT-PCR) using a high-throughput process that allows us to analyze more samples vs. more miRNAs in a very short time ^22^. Lastly, unlike hybridization-based microarray profiling techniques, qRT-PCR is considered the gold standard for RNA expression and does not require further confirmation analysis. Using this platform, a complete analysis of circulating whole blood, cellular, and cell-free miRNA was performed. The relevance of these findings to coronary disease was determined by measuring plasma miRNA expression in patients presenting for coronary angiography. The findings suggest that the distinct patterns of miRNA expression in components of whole blood may reflect specific patterns of disease.

## Results

### miRNA Profile in Human Plasma, Serum, Platelets, Mononuclear Cells and Whole Blood

There is minimal information defining miRNA expression in human blood and a complete screen of all known miRNAs has never been reported due to the limitations of current arrays and the cost of extensive qRT-PCR screening. Using blood samples from normal subjects and high-throughput qRT-PCR, the miRNA expression profile was determined for whole blood, isolated platelets, mononuclear cells, plasma and serum from five healthy subjects. Of the 852 miRNAs measured (Supplemental Table 1), the distribution of circulating miRNA that were most abundantly expressed in plasma and many of the blood derived sources is shown in Table 1. Gene expression is listed as cycle threshold value (Ct) consistent with RT-PCR-based data. Because the Ct values are listed, higher gene expression is reflected by a lower number. A complete list of miRNA expression for all sources is shown in Supplemental Table 2 (mean Ct value) and Supplemental Table 3 (mean delta Ct, accounting for housekeeping gene hsa-RNU1A-1). Unlike mRNA expression, it is currently unclear if miRNA data is more reliable when normalized with a housekeeping gene. This is especially germane for the cell-free plasma samples where a fixed volume was used and gene expression does not need to be normalized for cell count.

**Table 1.** Expression of miRNAs from whole blood, isolated platelets, mononuclear cells, plasma, and serum from healthy subjects (mean Ct values has shown; higher value indicates lower expression).

**miRNA Name**	**Plasma**	**PBMCs**	**Platelets**	**Whole Blood**	**Serum**
hsa-miR-1234	14.68	14.13	13.34	15.08	13.22
hsa-miR-641	15.20	14.66	14.43	15.08	15.50
hsa-miR-124a	15.63	17.54	17.25	18.59	18.25
hsa-miR-933	15.64	12.37	13.99	12.30	14.66
hsa-miR-7-2*	15.87	16.30	17.58	16.42	18.38
hsa-miR-323-3p	16.53	15.06	15.26	15.08	15.51
hsa-miR-631	17.34	15.96	16.43	16.43	17.01
hsa-miR-566	17.81	15.56	19.99	15.47	17.36
hsa-miR-1185	17.97	N/A	N/A	N/A	N/A
hsa-miR-766	17.98	13.70	15.76	13.94	16.55
hsa-miR-625*	18.03	20.71	19.75	20.90	18.80
hsa-miR-1228	18.06	14.63	17.15	16.36	18.07
hsa-miR-628	18.17	18.10	17.29	18.13	17.71
hsa-miR-601	18.38	15.22	17.46	15.73	18.85
hsa-miR-1281	18.78	16.72	16.65	16.75	17.14
hsa-miR-636	18.94	15.46	17.50	15.47	18.54
hsa-miR-657	19.04	19.11	17.50	19.39	18.50
hsa-miR-33b*	19.48	18.82	21.05	18.80	20.59
hsa-miR-553	19.51	N/A	N/A	N/A	N/A
hsa-miR-148a*	19.76	17.90	20.00	18.05	20.47
hsa-miR-637	20.09	17.88	18.08	18.25	19.65
hsa-miR-375	20.24	18.52	18.99	17.80	19.35
hsa-miR-129-3p	20.24	20.78	20.18	20.44	20.57
hsa-miR-99b	20.31	17.33	19.18	18.73	19.49
hsa-miR-380-5p	20.33	15.62	17.80	16.05	18.00
hsa-miR-508-5p	20.36	23.27	N/A	24.04	N/A
hsa-miR-1178	20.36	21.76	20.42	23.43	20.10
hsa-miR-92a	20.54	12.14	12.23	8.94	20.42
hsa-miR-888	20.55	20.25	18.76	20.30	18.72
hsa-miR-525*	20.59	24.62	N/A	N/A	N/A
hsa-miR-875-3p	20.63	18.91	20.36	18.98	20.64
hsa-miR-876-5p	20.64	20.37	22.90	21.25	20.37
hsa-miR-630	20.74	18.22	18.06	18.55	21.05
hsa-miR-519e	20.79	24.38	N/A	24.63	N/A
hsa-miR-632	20.84	16.96	19.90	17.08	20.58
hsa-miR-1301	20.86	19.18	19.74	19.85	20.22
hsa-miR-654	20.87	16.66	19.45	16.88	19.89
hsa-miR-548a-5p	20.90	N/A	N/A	N/A	N/A
hsa-miR-877*	21.16	19.21	21.59	19.60	21.38
GAPDH	21.17	14.52	17.52	15.06	22.86

miRNA results and demographic data. miRNA results and demographic data.

List of all miRNA assays. All miRNAs measured in whole blood, platelets, peripheral blood mononuclear cells (PBMCs) and plasma.

Unique distribution of miRNAs expressed in plasma, peripheral blood mononuclear cells (PBMCs), platelets, whole blood and serum. Average Ct values shown. Expression of miRNA measured in whole blood, platelets, peripheral blood mononuclear cells (PBMCs) and plasma. Ct value, higher number indicates lower expression.

Unique distribution of miRNAs expressed in plasma, peripheral blood mononuclear cells (PBMCs), platelets, whole blood and serum. Average delta Ct values shown, housekeeping gene Hs-RNU1A-1 used to calculate delta Ct. Quantitative expression of miRNA measured in whole blood, platelets, peripheral blood mononuclear cells (PBMCs) and plasma. Delta Ct value; values have been normalized using the housekeeping miRNA U44.

Expression of plasma miRNAs in patients with ≥ 70% CAD, patients with ≤ 70% CAD, and normal arteries. Data are expressed as mean delta Cts and standard deviation. Expression of plasma miRNAs in patients with 1) ≥70% CAD, 2) patients with

One-hundred and ninety four miRNAs were not expressed in any of the blood-based samples. Peripheral blood mononuclear cells (PBMCs) contained the highest number of miRNAs (658) followed by whole blood (609), platelets (448), serum (178) and plasma (147). As the abbreviated list shown in Table 1 demonstrates, while there is consistency between the groups, some miRNAs are more highly expressed in select sources. Sixty miRNAs were expressed in all five components. As the precise source of miRNA in plasma is not yet known, there was particular interest in comparing the expression between plasma and cellular miRNA patterns. Based on the significant overlap between the groups, it is difficult to determine the source for many of the specific plasma-derived miRNAs. It is not clear from the current data which miRNAs have a non-blood-based cellular source. Interestingly, miR-1185 and miR-548a-5p were much more abundantly expressed in plasma as compared to PBMCs or platelets, or only expressed in plasma. Although an interesting observation, the source of these two miRNAs cannot be determined from this data. In addition, some genes were expressed in platelets or PBMCs and not whole blood. This is likely due to the greater dilution used with PAXgene tubes and the loss of measurable expression of less abundant miRNAs.

### Plasma miRNA expression in patients with and without significant coronary disease

**Figure 1.**
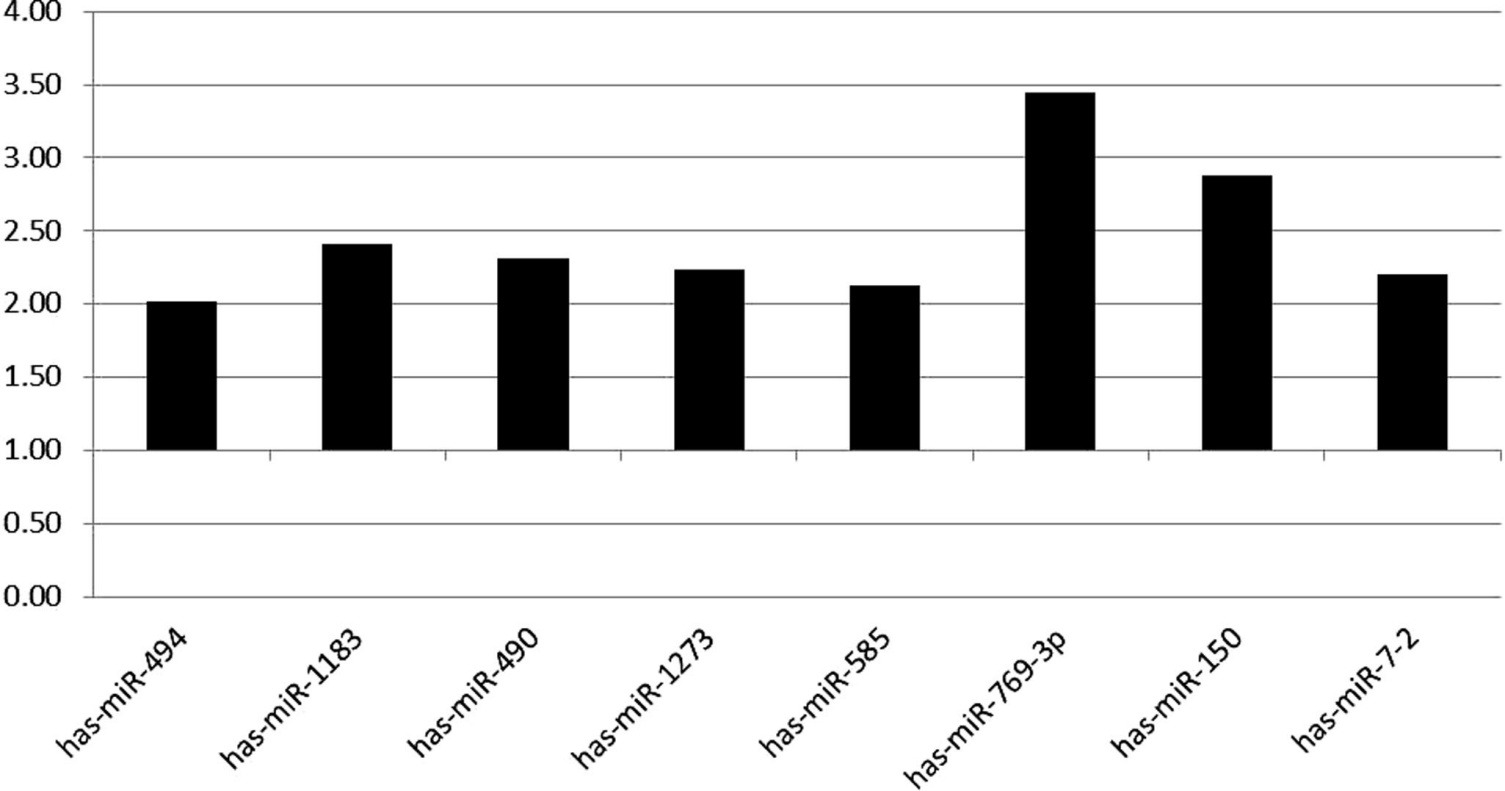
Fold change in plasma miRNA expression between in two groups. miRNAs shown had expression increased >2-fold between the groups; 1) ≥70% coronary stenosis of any coronary artery or 2) < 70 coronary stenosis.

The initial hypothesis of this study was that patients with significant coronary disease would have altered expression of plasma miRNA. Patients were divided into two groups (Table 2); 1) ≥70% coronary stenosis of any coronary artery or 2) <70 coronary stenosis. There were notable differences in expression of some of the miRNAs between these two groups (Table 3). By direct comparison, several plasma miRNAs were found to have over 2-fold increased expression in patients with significant coronary disease (≥70%) as compared to those with minimal coronary disease or normal coronary arteries (Figure 1). Initial statistical analysis demonstrated that anti-hypertensive therapy, smoking, and lipid lowering therapy have a positive association with coronary artery status (Table 2). Increased expression of miR-494, miR-769-3p and miR-490-3p was associated with ≥70% coronary stenosis. Next, the six variables were fit into a logistic regression analysis. As seen in Table 4, anti-hypertensive therapy, smoking and miR-769-3p were significantly associated with the coronary status.

**Table 2.** Characteristics of catheterization sample participants. Continuous measures, mean ± S.D., or number and percent with stated characteristic.

	**Non CAD** **Group (<70 CAD) n 49**		**CAD** **Group (≥70 CAD) n 34**	
	**Mean**	**SD**	**Mean**	**SD**
Age	56.2	11.3	61.3	10.6
Male	28.0		27.0	
Female	22.0		7.0	
Hypertension	21		27	
Smoking	8		19	
Lipid Treatment	6		16	
Diabetes	10		7	
Family History	14		11	
Antihypertensive Therapy	13		28	
Hormone Replacement Therapy	13		10	
Glucose	129.3	71.3	135.1	76.2
Total Cholesterol	169.9	36.6	168.4	40.5
HDL	39.5	8.8	36.5	12.5
LDL	99.2	29.5	94.4	34.0
Triglyceride	171.6	108.2	182.6	100.4

**Table 3.** Expression of plasma miRNAs in patients with ≥70% CAD as compared to patients with <70% CAD or normal arteries. Data is expressed as the mean delta Ct (Normalized to the RNU1A) and standard deviation. A lower number indicates increased miRNA expression. Bold valuesones are associated with CAD. More explanation in Table 5.

	**Non CAD Group**		**CAD Group**	
	**Mean** **Delta Ct**	**SD**	**Mean** **Delta Ct**	**SD**
hsa-miR-127	4.50	1.47	3.76	1.81
hsa-miR-129-3p	5.83	1.25	4.99	1.86
hsa-miR-532-3p	4.92	1.74	4.07	2.12
hsa-miR-433	5.65	1.32	4.90	1.80
**hsa-miR-494**	**5.12**	**1.32**	**4.11**	**2.16**
hsa-miR-375	-1.71	1.52	-2.64	1.99
hsa-miR-449	7.15	1.55	6.64	1.91
hsa-miR-323-3p	-7.48	2.21	-8.05	1.94
hsa-miR-1207-5p	5.00	2.30	4.08	2.61
hsa-miR-615-5p	5.02	1.23	4.25	1.74
hsa-miR-532	4.85	1.74	4.12	2.25
hsa-miR-99b	-1.83	1.31	-2.64	1.99
hsa-miR-1180	5.25	1.47	4.41	1.73
hsa-miR-1224-5p	5.12	4.32	4.76	2.72
hsa-miR-574	5.01	1.50	4.33	2.02
hsa-miR-92a	6.54	1.93	5.64	2.16
hsa-miR-1183	7.32	4.91	6.05	5.59
hsa-miR-92b	5.18	1.68	4.26	1.92
**hsa-miR-490-3p**	**3.34**	**1.33**	**2.13**	**2.02**
hsa-miR-1253	4.14	3.64	4.10	1.94
hsa-miR-1273	3.28	3.99	2.12	3.26
hsa-miR-1301	0.66	3.21	0.68	1.97
hsa-miR-1972	3.91	2.00	3.01	2.16
hsa-miR-345	3.91	1.52	3.29	2.07
hsa-miR-383	5.78	0.61	5.35	1.46
hsa-miR-489	-0.37	1.14	-0.62	1.29
hsa-miR-202	2.82	0.65	2.67	1.19
hsa-miR-373	3.98	0.53	3.76	1.15
hsa-miR-220	2.45	1.55	1.86	2.09
hsa-miR-320a	4.11	1.02	4.03	0.98
hsa-miR-432	4.25	0.86	4.11	0.98
hsa-miR-483-3p	3.57	0.46	2.66	5.67
hsa-miR-339-3p	5.15	0.73	4.82	1.19
hsa-miR-483-5p	-1.29	0.52	-1.35	0.61
hsa-miR-487a	4.35	0.45	4.10	1.00
hsa-miR-548b-5p	5.40	1.74	4.44	2.66
hsa-miR-502	1.56	1.20	0.89	1.94
hsa-miR-631	-3.98	1.63	-4.52	2.06
hsa-miR-555	1.81	1.62	1.29	2.00
hsa-miR-641	-5.04	1.91	-5.82	2.44
hsa-miR-566	5.56	1.78	4.80	2.13
hsa-miR-628	1.36	1.63	0.75	1.94
hsa-miR-585	5.60	1.85	4.51	2.55
hsa-miR-637	-1.81	1.65	-2.37	2.03
hsa-miR-767-3p	4.32	1.57	3.91	1.80
**hsa-miR-769-3p**	**4.39**	**2.96**	**2.61**	**3.42**
hsa-let-7b	4.43	8.29	4.25	4.00
hsa-miR-1914	4.80	4.82	5.45	2.41
hsa-miR-149	4.40	5.51	4.75	2.40
hsa-miR-150	2.04	2.85	0.51	2.70
hsa-miR-33b	0.87	2.02	-0.07	2.22
hsa-miR-7	-3.73	1.84	-4.87	3.82

**Table 4.** Logistic regression analysis using demographic characteristics and significantly differentially expressed miRNAs.

	**Estimate**	**Std.** **Error**	**z value**	**Pr (>|z|)**
(Intercept)	-1.63	1.12	-1.44	0.14
Antihypertensive Therapy	2.86	0.77	3.70	0.000213 ***
Smoking	2.44	0.77	3.16	0.001568 **
Lipid Lowering Therapy	0.74	0.73	1.01	0.30
hsa-miR-494	0.05	0.27	0.20	0.83
hsa-miR-490-3p	-0.30	0.31	-0.96	0.33
hsa-miR-769-3p	-0.23	0.11	-1.98	0.047044 *

### Plasma miRNA expression: significant coronary disease vs. disease-free

**Figure 2.**
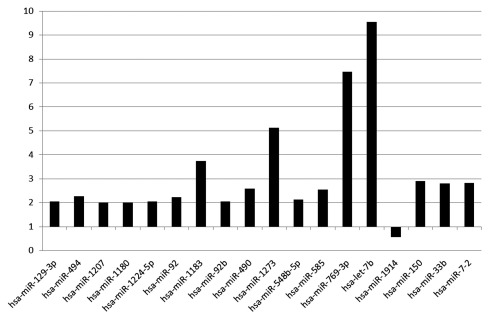
Fold change in plasma miRNA expression between three groups. miRNAs shown had expression increased >2-fold or decreased <0.5-fold for the groups; 1) patients with CAD; at least one of the coronary arteries have ≥70% occlusion; 2) patients with atherosclerosis but not clinically significant CAD; coronary occlusion 1%–<70%; and 3) coronary atherosclerosis-free, patients with no angiographically documented coronary artery stenosis.

We initially determined whether presence of significant coronary disease, as defined by ≥70% stenosis is associated with specific miRNA expression. A secondary question is whether presence/absence of coronary disease is associated with miRNA expression. To study this, patients were placed into one of three groups; 1) patients with CAD; at least one of the coronary arteries have ≥70% occlusion; 2) patients with minimal CAD; coronary occlusion 1%–<70%; and 3) coronary atherosclerosis-free, patients with no angiographically documented coronary artery stenosis. Because the numbers are small, a simple (non-statistical) comparison was made to determine trends. As seen in Figure 2 and supplemental table 4, 18 miRNAs had a varied expression pattern between group 1 and group 3. Seventeen miRNAs were upregulated at least 2-fold with only miR-1914 downregulated 0.5-fold. Interestingly, most miRNAs demonstrated a dose response with the greatest expression in patients with the most coronary disease and the least expression in disease-free patients (Supplemental table 4).

### miRNA target predictions

Currently, the capacity to measure miRNAs far outpaces our ability to understand their function in a given tissue. However, to better understand the potential significance of our findings, we conducted analyses that predict targets of the miRNAs that were up- or downregulated in coronary disease using two methods; current publications ( www.ncbi.nlm.nih.gov/pubmed/) and TARGETSCAN 5.1 ( www.targetscan.org). Using these methods, results for miRNA targets varied between 3 to 369 targets per miRNA when identified. With the TARGETSCAN search, target genes for individual miRNAs were identified using the context score for specific sites within genes. The context score is the sum of site-type contribution, 3’ pairing contribution, local AU contribution, and position contribution. The lower the context score indicates the most highly predicted targets for each miRNA. By TARGETSCAN search, miRNAs miR-1914 and miR-7-2, had no target gene identified.

Detailed predictions for the miRNAs found to be significant in CAD are shown in Table 5. In broad terms, some miRNAs appear to target transcription factors, growth factors, cytokine regulation, transmembrane proteins, signal transduction pathways, and epigenetic pathways such as histone acetylases. Prediction results for specific miRNAs in TARGETSCAN include the following: miR-129-3p and miR-494 target HMGCS1; miR-150 targets MMP14; miR-150, and 92b target MMP16. Additionally, miR-1207 appears to target energy metabolism (most likely involving glucose metabolism).

**Table 5.** MiRNA predictions for target genes associated with significant coronary disease using TARGETSCAN. Individual miRNAs target genes identified as significantly different between patients with and without significant coronary disease were selecting using the context score, a sum of; site-type contribution, 3’ pairing contribution, local AU contribution, and position contribution. The lowest scores show highest predictions.

**Target gene**	**Gene name/miRNA Name**	**Total Context Score**
hsa-miR-494*		
SOCS6	Suppressor of cytokine signaling 6	-1.06
HMGCS1	3-hydroxy-3-methylglutaryl-Coenzyme A synthase 1 (soluble)	-0.88
COL6A6	Collagen type VI alpha 6	-0.64
BCAP29	B-cell receptor-associated protein 29	-0.53
SCARB2	Scavenger receptor class B, member 2	-0.52
IGF1R	Insulin-like growth factor 1 receptor	-0.31
F11R	F11 receptor	-0.41
hsa-miR-490-3p*		
LEP	Leptin	-0.32
IL1RAP	Interleukin 1 receptor accessory protein	-0.31
CD63	CD63 molecule	-0.29
TGFBR1	Transforming growth factor, beta receptor I (activin A receptor type II-like kinase, 53kDa)	-0.21
ADIPOR2	Adiponectin receptor 2	-0.2
MEF2D	Myocyte enhancer factor 2D	0
hsa-miR-769-3p*		
FAM130A1	Family with sequence similarity 130, member A1	-0.4
SHPK	Sedoheptulokinase	-0.39
KCNA5	Potassium voltage-gated channel, shaker-related subfamily, member 5	-0.35
MARCKS	Myristoylated alanine-rich protein kinase C substrate	-0.25
PBRM1	Polybromo 1	-0.24
ISOC2	Isochorismatase domain containing 2	-0.23
MYCL1	V-myc myelocytomatosis viral oncogene homolog 1, lung carcinoma derived (avian)	-0.23
KPNA6	Karyopherin alpha 6 (importin alpha 7)	-0.2
KDSR	3-ketodihydrosphingosine reductase	-0.17
COLEC12	Collectin sub-family member 12	-0.15
TSPAN1	Tetraspanin 1	-0.15
PIM2	Pim-2 oncogene	-0.11
KIAA1128	Family with sequence similarity 190, member B (FAM190B)	-0.07

We also analyzed the miRNAs that were unique to plasma, miR-1185-1 and miR-548a-5p-1. While many potential targets were identified, these miRNAs were predicted to target genes involved in controlling transcription factors, as well as several growth and cell cycle components.

## Discussion

MicroRNAs (miRNAs) are short regulatory RNAs that participatein the control of approximately 30% of all protein-coding genes ^4– 6^. The expressionof many miRNAs is usually specific to a tissue or developmentalstage, and the miRNA expression pattern is altered during theprogression of several diseases ^7, 8^. Most miRNAs are transcribed by RNA polymerase II from individual miRNA genes, from introns of protein coding genes, or from polycistronic transcripts that often encode multiple related miRNAs ^4, 23^. Although miRNAscan guide mRNA cleavage, the basic function of miRNA is to mediateinhibition of protein translation ^1, 7, 24– 27^ through miRNA-inducedsilencing complexes (miRISCs). The guiding strand of miRNA ina miRISC interacts with a complementary sequence in the 3’-untranslatedregion (3’-UTR) of its target mRNA by partial sequence complementarities, resulting in translational inhibition ^1, 7^.

In this study, the distribution of miRNA expression in whole blood, platelets, PBMCs plasma and serum showed significant overlap. Of particular interest are the nucleus-lacking platelet and the cell-free plasma expression levels. A primary question is why platelets would have miRNA? Platelets are produced in the bone marrow from megakaryocytes as cytoplasmic fragments without genomic DNA ^28^. Platelets, however, retain a small amount of megakaryocyte-derived messenger RNAs (mRNAs) that have recently been suggested to be of physiological significance. Platelets can respond to physiological stimuli at the levels of protein translation and mRNA splicing ^29, 30^. There are few published studies describing platelet miRNAs ^13^. In this study, cells of hematopoietic lineage were described to have a limited number of miRNAs (this study only tested for 19 miRNAs) and functionality was not shown. Interestingly, our data demonstrate that platelets express nearly the same number of miRNAs as PBMCs. The number of miRNAs in PBMCs is slightly less than in whole blood with the reason likely being dilution of low abundant PBMC miRNAs in whole blood.

In limited numbers, miRNAs have been detected in cell-free human plasma preparations ^14^. They have been found to be stable and protected from endogenous RNase activity ^14^. In addition, levels of a specific miRNA ( *miR-141*) can distinguish patients with prostate cancer from healthy controls. In our analysis, we found moderate to high levels of expression of miRNAs in plasma in both normal subjects and patients with coronary disease, albeit in lower numbers as compared to platelets and PBMCs. What these data do not provide is the specific source of circulating miRNAs. This is a fundamental and fascinating question. They are believed to arise from three potential mechanisms: apoptosis, cellular activation with release of protrusions, and microsome/microvesicle formation. For example, Mitchell et al. found miR-141 differentially expressed in plasma in microsomes of prostate cancer patients ^14^. It is possible that the miRNAs detected by our measurements in plasma or blood could be derived from endothelial cells or the atherosclerotic plaque itself. Further fundamental experiments are needed to answer this question. Recently, it has been shown that HDL particles deliver miRNAs ^31^. Additionally, Wang et al. reported that plasma and whole blood miR-133 and miR-328 levels are increased in AMI patients ^32^.

By the current data, we cannot assign precise punitive targets; however, specific bioinformatics approaches have been developed to predict miRNAs present in the genome of different organisms. These techniques are based on the observation that transcripts are usually highly conserved between related species and produced from precursor transcripts of similar size and structure. Using these bioinformatics approaches and the limited information available in the literature, we assembled potential target genes for the miRNAs expressed in significantly different amounts between patients with and without ≥70% coronary disease (Table 5). The list included a diverse range of functional and structural genes. This includes leukocyte and platelet recruitment to the atherosclerotic tissues, matrix reorganization, foam cell formation, growth/proliferation, and angiogenesis. However, these predictions do not provide definitive targets and additional basic studies are needed to provide clearer mechanistic information.

Also unique to our study is the specific method of measurement we used, which allowed for flexibility in adding newly discovered miRNAs, the use of small volumes, and high-throughput methods for qRT-PCR. Currently, there are several miRNA microarray products available that measure fewer miRNAs and some consist of older versions of the Sanger miRBase Sequence Database. Using the universal cDNA reaction feature of miScript provided the ability to profile all miRNAs with one cDNA reaction. Unlike the hybridization-based microarray profiling techniques, by coupling the miScript and Biomark Systems, confirmation analysis was not required for individual miRNAs. However, there are important limitations of our study. Despite the expansive miRNA survey for blood components, we cannot define the specific source for the plasma miRNA nor its eventual destination. Our study of coronary patients was limited by only being able to evaluate plasma miRNA, as other blood components were not available to us. In addition, while the miRNA data provided from these patients are unique, the numbers are still small making further analysis based on any subgroup statistically unfeasible.

In summary, miRNAs are small RNAs that play an important role in the negative regulation of gene expression by suppressing protein translation and have been detected in cell-free plasma and have been related to select diseases. By examining all measurable miRNAs, we defined the relative expression in blood components and find significant expression in platelets, PBMCs, whole blood, plasma and serum. By comparing plasma miRNA expression in patients with coronary disease, we begin to define specific miRNAs that are altered and provide potential targets that influence atherosclerosis.

## Materials and methods

### Sample procurement

This study was approved by Mersin University Ethical Committee (06/05/2009, #6/144) and written consent was obtained from the subjects to test the hypothesis of whether coronary occlusion of ≥70% is associated with increased plasma miRNA expression levels. Upon enrollment, a study coordinator identified the presence of the following risk factors: (1) age, (2) male sex, (3) clinical history of diabetes, (4) clinical history of hypertension, (5) cigarette smoking, (6) clinical history of hypercholesterolemias, and (7) family history of coronary disease. Coronary angiograms were analyzed off-line in a blinded fashion with the use of digital calipers to measure stenosis severity, and stenosis was defined as a dichotomous variable: if a stenotic lesion was ≥70%, that vessel was counted as stenosed. The presence or absence of stenotic disease was also noted. Patients were ranked as having 0- to 3-vessel disease (number of coronary arteries with detectable atherosclerotic disease). For each patient, K _3_EDTA arterial blood (5 mL) was collected just prior to coronary angiography. Blood samples were centrifuged (3,000 g) and 400 µl plasma samples were stored at -80°C until RNA isolation.

In a separate smaller study, blood was obtained from healthy consented volunteers (n=5; 3 female, 2 male, average age=45) at Boston University School of Medicine as previously described ^33^. The study was approved by Boston University IRB and written consent obtained from the subjects. All subjects were free of medications or supplements, and had no history of hypertension, diabetes, smoking, or hyperlipidemia. Blood was collected into PAXgene RNA tubes (Becton Dickinson, Franklin Lakes, NJ) for whole blood, into CPT tubes (Becton Dickinson) for peripheral blood mononuclear cells (PBMCs), into citrate tubes for platelets and plasma, and empty tubes for serum. Isolated platelets were prepared as previously described.

**RNA isolation:** Total RNA including miRNAs was isolated from 200 µl plasma samples using miRVana Paris Kit (Ambion, Austin, TX). The RNA samples were stored at -80°C until cDNA conversion.

**cDNA conversion:** Isolated RNA samples were converted to cDNA using miScript Reverse Transcription Kit (Qiagen, Germantown, MD). The RNA was converted to cDNA using the following conditions: 37°C for 60 min, 95°C for 5 min, and 4°C hold until further processing or storage. cDNA samples were kept at -80°C until PCR analysis.

**Pre-amplification:** Prior to PCR, cDNA samples were pre-amplified using Taqman PreAmp Master Mix (Applied Biosystems, Foster City, CA). PreAmp Master Mix and 0.2x Primers were added to the cDNA samples and pre-amplified as follows: 95°C for 10 min once, 95°C for 15 sec, 55°C for 30 sec, 70°C for 4 min (final three steps repeated for 14 cycles).

**qRT-PCR:** Quantitative Real-Time PCR reactions (qRT-PCR) were performed using the high-throughput BioMark Real-Time PCR system (Fluidigm, South San Francisco, CA). Pre-amplified cDNA samples were diluted with 0.1mM EDTA in TE Buffer (1:5) and mixed with Power Sybr Green PCR Master Mix (Applied Biosystems), AmpliTaq Gold DNA Polymerase (Applied Biosystems) and Sample Loading Reagent (Fluidigm), then pipetted into sample inlets of Dynamic Array 96.96 chips (Fluidigm). Assay Loading Reagent (Fluidigm) and primers (Qiagen) were mixed and pipetted into assay inlets of Dynamic Array 96.96 chips. The IFC Controller HX (Fluidigm) was used to distribute primers and samples into chip reaction wells for qRT-PCR by microfluidic delivery. Gene expression experiments performed at Mersin and Gaziantep Universities in Turkey. The data were normalized using RNU1A1. Coefficient variations were less than 10% for almost all of the assays. Plasma volumes for all samples were constant (200 µl) and all following steps such as cDNA, PreAmplification and qRT-PCR had the same volumes always for all samples.

### Statistics

When examining miRNAs that had larger fold changes between the CAD groups ( Figure 1 and Figure 2) and those more highly expressed in the circulation using similar bioinformatics methods (such as targetscan), there were many miRNA targets that are known to control the processes important in the development of atherosclerosis (list not shown).

### Univariate and bivariate analysis

We initially summarized our data in different stratifications based on our outcome variables (coronary disease status). Next, we examined the bivariate relationship between the response variable and quantitative covariates using either two-sample t-test or Kruskal-Wallis test, where appropriate. Specifically, the t-test was conducted to test for a mean difference in quantitative demographic variables and miRNA expression level between two categories of coronary disease status. The pooled variance or the Satterthwaite’s method was used to estimate variance based on the equality of variance test. We employed Kruskal-Wallis test to determine whether there was any mean difference among groups in the scenario with three-category outcome variable.

### Multivariate analysis

Tree-based methods have been increasingly applied to biological research such as microarray data analysis and genome-wide association studies ^34, 35^. RandomForest is a flexible nonparametric approach, which consists of many decision trees from bootstrap samples. In this study, we constructed randomForest ^25^ in identifying relevant variables to our outcome variable using the randomForest package in R (2.10.1) ^36^. Furthermore, we also conducted logistic regression and multinomial logistic regression where appropriate ^37^.
