# Supplementary material for: The distribution of circulating microRNA and their relation to coronary disease
Source: F1000Res. 2012 Nov 19;1:50. [Version 1] doi: 10.12688/f1000research.1-50.v1 (PMC3752638; doi:10.12688/f1000research.1-50.v1)
Supplement: Peer review history

# Peer review: Referee response for version 1

**Published:** 2013-01-11

**Authors:** Jennifer Clancy

**Affiliations:** Genome Biology Department, John Curtin School of Medical Research, ANU College of Medicine, Biology & Environment, The Australian National University, Canberra, Australia

I would like to note that the manuscript could be improved by use of error bars in Figures 1 and 2 to display some measure of variance and that future studies of this type could utilise spike-ins prior to RNA extraction to normalise for variability in RNA extractions between samples.

I have read this submission. I believe that I have an appropriate level of expertise to confirm that it is of an acceptable scientific standard.

# Peer review: Referee response for version 1

**Published:** 2013-01-09

**Authors:** Terry Elton

**Affiliations:** Division of Pharmacology, OSU College of Pharmacy, Columbus, Ohio, USA

I have read this submission. I believe that I have an appropriate level of expertise to confirm that it is of an acceptable scientific standard.

# Peer review: Referee response for version 1

**Published:** 2012-11-26

**Authors:** Peter Sarnow

**Affiliations:** Department of Microbiology and Immunology, Stanford University, Stanford, CA, USA

I have read this submission. I believe that I have an appropriate level of expertise to confirm that it is of an acceptable scientific standard.
